# The cuticular wax composition and crystal coverage of leaves and petals differ in a consistent manner between plant species

**DOI:** 10.1098/rsob.230430

**Published:** 2024-05-29

**Authors:** Sverre Aarseth Tunstad, Ian D. Bull, Sean A. Rands, Heather M. Whitney

**Affiliations:** ^1^ School of Biological Sciences, University of Bristol, Bristol, UK; ^2^ Organic Geochemistry Unit, School of Chemistry, University of Bristol, Bristol, UK

**Keywords:** cuticular waxes, flower cuticle, plant–pollinator interactions, wax crystals

## Abstract

Both leaves and petals are covered in a cuticle, which itself contains and is covered by cuticular waxes. The waxes perform various roles in plants’ lives, and the cuticular composition of leaves has received much attention. To date, the cuticular composition of petals has been largely ignored. Being the outermost boundary between the plant and the environment, the cuticle is the first point of contact between a flower and a pollinator, yet we know little about how plant–pollinator interactions shape its chemical composition. Here, we investigate the general structure and composition of floral cuticular waxes by analysing the cuticular composition of leaves and petals of 49 plant species, representing 19 orders and 27 families. We show that the flowers of plants from across the phylogenetic range are nearly devoid of wax crystals and that the total wax load of leaves in 90% of the species is higher than that of petals. The proportion of alkanes is higher, and the chain lengths of the aliphatic compounds are shorter in petals than in leaves. We argue these differences are a result of adaptation to the different roles leaves and petals play in plant biology.

## 1. Introduction

Plant cuticular waxes consist of a variety of long-chained aliphatic compounds, such as alkanes and primary alcohols, as well as triterpenoids and other cyclic compounds [1]. Located at the very interface between the plant and the external environment, plant waxes play several roles, the most important of which is reducing water loss through the cuticle [2–4]. Most of this barrier is situated within the cuticle itself [5–9]. On top of the cuticle, there is a thin layer of epicuticular waxes. Sometimes, this layer is not visible owing to the presence of epicuticular crystals, which play different roles than the intracuticular waxes. These crystals show great structural and chemical diversities between species and plant organs [10]. They can alter the reflectance spectra of leaves and fruits [11–13], act as a self-cleaning surface [14,15], trap insects [16,17] and prevent nectar robbers from easily accessing flowers [18]. While the waxes of the cuticle as a whole often contain a large variety of different compounds, the wax crystals are usually composed of a single compound or closely related homologues [1]. Because these cuticular waxes make up the interface between the plant, its external environment, and any species that may visit the plant, their chemistry and ecology will have been shaped by the types of interactions that the plant experiences, and these interactions will differ between different areas of the plant.

Most studies of cuticular wax tend to focus on leaves (e.g. [10,19–21]) and, to a lesser extent, fruits (e.g. [6,22,23]). Agricultural interests are often the backbone of these studies, which means that water management, pest management and crop shelf-life are priorities (e.g. [24,25]), particularly with the cuticle surface having a direct impact on the longevity of fruit [6,26]. The cuticular waxes of flowers have been much less studied, and there are only a few species in which the full wax profile of floral cuticular waxes has been investigated. These include snapdragon (*Antirrhinum majus*) [27], *Petunia hydrida* cv. ‘Mitchell’ [28], *Arabidopsis thaliana* [29], *Hibiscus trionum* [30], as well as the epicuticular waxes of raspberry (*Rubus idaeus*) and hawthorn (*Crataegus monogyna*) [31]. Additionally, some plants have had both leaves and petals analysed, enabling direct comparisons between the different organs (table 1). These studies demonstrate differences in the overall wax load between organs, and most show a higher relative proportion of alkanes in petals, as well as shorter carbon chain lengths in the aliphatic compounds of petals compared to leaves.

**Table 1.** An overview of plants in which leaf and petal (or tepal) total wax composition have been analysed in the same study.

plant	organ	wax coverage (µg cm^−2^)	alkane chain length	permeance (m s^−1^)	percentage of alkanes	**study**
*Cistus albidus*	leaf petal	200 5	longer shorter	na na	9.4 31.2	[32]
*Cosmos bipinnatus^a^*	leaf petal	12.4 2.7	longer shorter	~1.0 × 10^–4^ 6.7 × 10^–5^ (abaxial) and 3.4 × 10^–5^ (adaxial)	32 16	[33]
*Taraxum officinale^a^*	leaf petal	7.14 37	na na	na na	trace 30	[34]
*Solanum tuberosum*	leaf petal	1.78 2.56	longer shorter	na na	52 35	[35]
*Vicia faba* ‘chenghu 10’	leaf petal	1.99 2.78	similar similar	na na	7 45	[36]
*Rosa chinensis* ‘movie star’ [*sic*]	leaf petal	6.7 13.2	longer shorter	1.9 × 10^–5^ 4.1 × 10^–5^	36.8 46.8	[37]
*Rosa chinensis* ‘tineke’ [*sic*]	leaf petal	5.8 4.9	longer shorter	1.8 × 10^–5^ 1.0 × 10^–4^	37.4 64.3	[37]
*Lilium* cv. ‘tiber’	leaf tepal	1.52 7.41	longer shorter	1.5 × 10^–5^ 1.3 × 10^–4^	44.1 33.0	[38]

^a^ Plants in which the real surface area was calculated by taking into account the cell shape of the plant surfaces.

The studies presented in table 1 suggest that waxes differ between leaves and petals, which makes sense given that flowers and leaves experience very different interactions with both the environment and any visitors to the plant. The total wax load is higher in some petals than their respective leaves, but the petal cuticles have a higher permeance than the leaves in three out of the four species in which it was investigated. Leaves may last a long time on a plant and will need to survive continuous and prolonged attacks from both herbivores and microorganisms; flowers, on the other hand, may be more ephemeral and need to balance avoiding attack with attracting pollinators. Furthermore, flowers present humid microenvironments [39], with transpiration across the petal surface [40] contributing to this humidity. We could therefore hypothesize that variations in the wax chemistry between petals and leaves reflect these different interactions, as is hinted at by the reduced petal alkane chain lengths seen in table 1. This may in turn impact the permeability of the cuticle by altering the ratio of amorphous to crystalline regions in the waxes comprising the transpiration barrier of petals as hypothesized by Riederer & Schneider [41].

In this study, we aimed to investigate whether the patterns found in previous studies with regard to lower total wax loads and shorter chain lengths in petals hold true when looking at a larger set of plants from across various plant orders. We did this by sampling a wide range of plant species and comparing the lipid composition of petal and leaf waxes while controlling for similarities owing to shared evolutionary history.

## 2. Results

### 2.1. Scanning electron microscopic imaging

We found a range of crystal shapes in the leaves, which we classified according to Barthlott *et al*. [10]. There were large differences in the crystal coverage of leaves and petals. Twenty-three out of 49 leaves and 2 out of 49 petals had wax crystals on both surfaces. Twenty-one leaves and 46 petals had no crystals on either surface. Five leaves and one petal had crystals on one side only. In cases where leaves had crystals on one side only, the crystals were all found on the upwards facing (adaxial) side.

### 2.2. Total wax coverage

**Figure 1.**
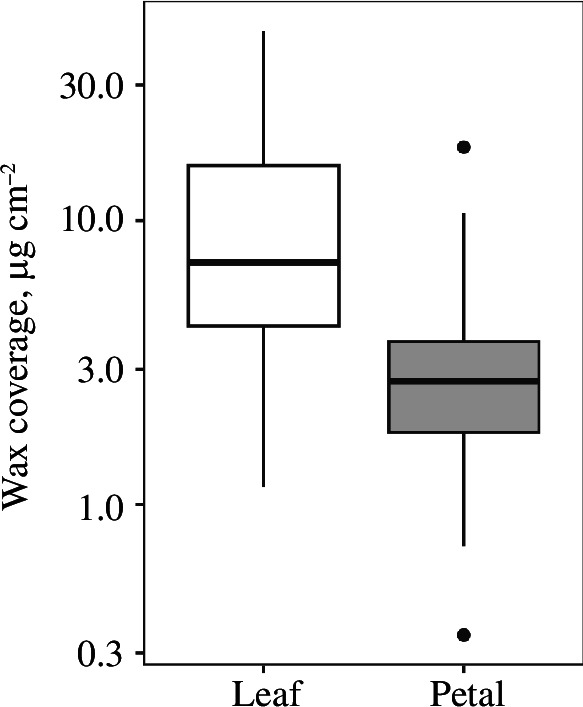
Total wax loads of leaves and petals. The median is represented by the central line in each box, which contains the 25th to 75th percentiles of the values. The black whiskers indicate the 5th and 95th percentiles, and wax loads beyond these upper and lower bounds are considered outliers, marked with dots.

Leaves had a higher mean wax coverage than petals (leaves: 12.12 ± 11.09 µg cm^−2^, petals: 3.33 ± 2.81 µg cm^−2^; *t*_46_ = 8.86, *p* < 0.001, λ = 0; figure 1; electronic supplementary material, figure S1). Considering leaves alone, species with crystal-covered leaves had a higher mean wax coverage on the leaves than crystal-free species (*t*_47_ = 5.19, *p* < 0.001, electronic supplementary material, figure S2), while the wax coverage on the petals when crystals were present was greater than when crystals were absent (*t*_47_ = 2.25, *p* = 0.029; electronic supplementary material, figure S2), although we suggest caution in interpreting this latter result as only three species had crystals present on the petals. We did not calculate the true surface area caused by differences in cell shape but assessed the amount of surface underestimation from the amount of shrinkage artefacts on the scanning electron microscope (SEM; electronic supplementary material, table S1). The pavement cells on the leaves were largely flat, and there appeared to be little surface underestimation. There were a larger number of conical cells in the petals, and the surface underestimation was therefore bigger in the petals.

### 2.3. Wax composition

The wax composition of the leaves was not evolutionarily constrained (λ = 0.171, 95% confidence interval (CI) 0.067–0.283, but with the calculated value of λ, only greater than 94% of the values generated during permutation testing; electronic supplementary material, figure S3*a*), but the wax composition of the petals showed moderate constraint owing to evolutionary history (λ = 0.296, 95% CI 0.159–0.435, with the calculated value of λ greater than 99% of the values generated during permutation testing; electronic supplementary material, figure S3*b*).

**Figure 2.**
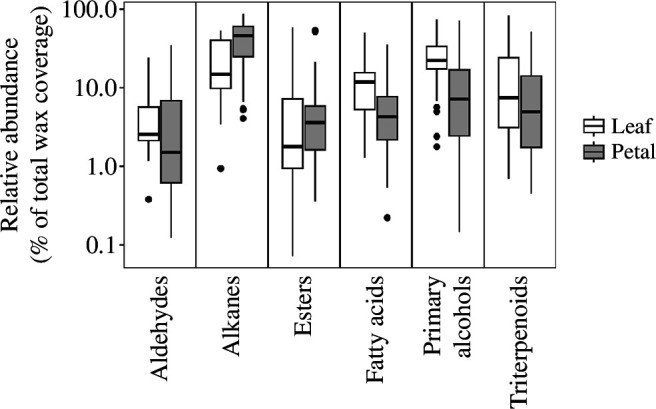
Relative abundance of the most common compound classes on leaves and petals (crystal-free samples only).

While no compound classes were found exclusively in leaves or petals, the wax composition of leaves and petals differed in relative composition. Within species, the wax on leaves tended to be composed of a larger amount of aldehydes, esters, fatty acids, and primary alcohols when compared to the petals (aldehydes: *t*_46_ = 4.20, *p* < 0.001, λ= 0.083; esters: *t*_46_ = 2.07, *p* = 0.044, λ= 0; fatty acids: *t*_46_ = 2.18, *p* = 0.034, λ= 0.427; primary alcohols: *t*_46_ = 5.08, *p* < 0.001, λ= 0; figure 2), but leaf waxes had a lower proportion of alkanes when compared with petals of the same species (*t*_46_ = −4.59, *p* < 0.001, λ= 0; figure 2). There were no directional differences for triterpenoids (*t*_46_ = 0.56, *p* = 0.577, λ= 0).

**Figure 3.**
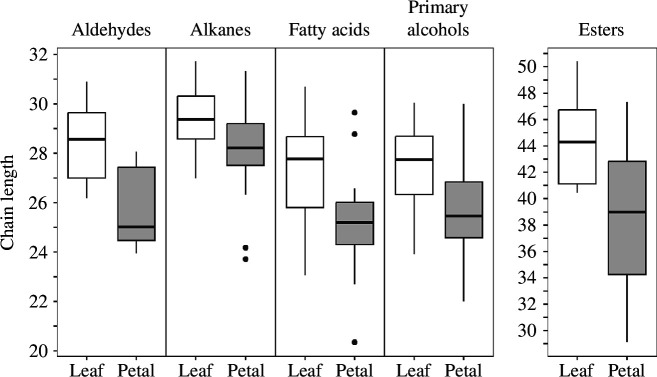
Average chain lengths of the most common aliphatic compounds, grouped by organ, indicating that the chain lengths are consistently shorter in petals than in leaves. Only plants in which the compounds were found in both leaves and petals were included in the comparison.

Considering only cases in which the same compound classes were present in both leaves and petals of the same plant, the average chain length (ACL) of the major long-chain compounds (aldehydes, alkanes, esters, fatty acids and primary alcohols) were shorter in the petals than in the leaves (aldehydes: *t*_8_ = 4.74, *p* = 0.001, λ = 0; alkanes: *t*_42_ = 5.23, *p* = 0.001, λ = 0; esters: *t*_5_ = 3.56, *p* = 0.016, λ = 0; fatty acids: *t*_17_ = 4.18, *p* < 0.001, λ = 0, *t*_43_ = 4.23; primary alcohols: *t*_43_ = 4.23, *p* < 0.001, λ = 0.251) (figure 3; electronic supplementary material, figure S4).

## 3. Discussion

Leaves had a higher total wax coverage than the corresponding petals in 44 out of 49 species. This most likely reflected the presence and absence of wax crystals on leaves and petals, which is supported by findings reported in previous publications correlating crystal coverage to higher wax loads [1]. The same correlation was found in our data when looking at the total wax composition grouped by crystal coverage (electronic supplementary material, figure S2). The higher proportion of alkanes in petals observed in previous studies (table 1) was confirmed here and was mirrored in lower proportions of all other compounds in the petal cuticles.

The ACL of aliphatic compounds in leaves is higher than in petals. Shorter aliphatic compounds could in theory lead to less effective transpiration barriers [41]. There is some evidence that petals are frequently leakier than leaves. Besides the papers listed in table 1, it was shown in a recent study that petal cuticles were leakier than those of leaves in 74 out of 101 plant species investigated [42].

The difference in wax crystal coverage on leaves and petals is most likely to be a result of the varying selection pressures to which leaves and petals are subjected. Whereas leaves are optimized for photosynthesis and longevity, the role of petals in animal-pollinated plants is not only to attract pollinators but also to ensure successful pollination. This includes facilitating pollinator attachment. Bumblebees avoid flowers that are difficult to physically handle [43]. Wax crystals are known to prevent insect attachment, as demonstrated by pitcher plants’ and *Macaranga* ant plants’ use of crystals to trap or prevent all but select ant species from adhering to their stems, respectively [16,44,45]. Such crystals might be selected against in a setting where pollinator attachment is key to successful pollination. It is also possible that the pattern is a result of the reduced need to protect petals from pathogens and environmental stressors over long periods of time. While leaves last an entire growth season, flowers tend to be open for much shorter amounts of time, and an investment in thick wax crystal coverage might not be beneficial when the expected duration of the structure is short. Looking at crystal coverage as a function of the average lifespan of the flowers might provide information to this end.

Whether the shorter chain lengths in the petals are an adaptive feature, a result of a reduced need for long chains, or of developmental restraints needs to be explored further. A highly impermeable cuticle might not be a problem in petals as it would in leaves, as plants deal with drought stress differently in the different organs. Here, we found that the composition of the waxes is likely to be relatively conserved between closely related species.

In this paper, we have expanded our understanding of floral cuticular wax composition. We show that there are recurring differences between petal and leaf cuticular wax, and these differences are found across the phylogenetic tree. We argue that they reflect the roles flowers play in the plant life cycle. Following on from the findings in our paper, it will be interesting to investigate whether the cuticular composition of petals is shaped by pollinator groups, and whether environmental differences lead to changes in floral cuticular wax composition and properties.

## 4. Methods

### 4.1. Specimens

The plant material was collected on and around the University of Bristol campus. Leaves and petals were collected using clean metalware and stored in furnace tin foil until wax extraction. The wax extraction took place within an hour after detachment from the plant. The plants in the study are listed in the electronic supplementary material, table S2.

### 4.2. Collection of waxes and chemical analysis

All surfaces used for quantification were imaged using a digital camera, and the surface area was measured in Figi [46]. The surface area extracted per plant organ ranged from 2 to 44 cm^2^, owing to the large variation in leaf and petal sizes. Whole wax mixtures were obtained from leaves and petals by submerging them for 30 s in a test tube containing 5 ml of analytical grade chloroform held at 50°C and 10 μg of tetracosane (internal standard). The solutions were then filtered using a grade 100 filter paper (Fisherbrand) into a 7 ml sample vial, and solvent evaporated under a stream of nitrogen. The samples were then stored at –20°C. On the day of analysis, each sample was resuspended in 1 ml of CHCl_3_ and sonicated for 10 min. Following this, 100 μl was aliquoted and subjected to the following treatment: the solvent was evaporated under a stream of nitrogen, and 30 μl of *N*,*O*-bis(trimethylsilyl)trifluoroacetamide (BSTFA) containing 1% trimethylchlorosilane (TMCS) was added for silylation of the hydroxy and carboxyl groups, in order to reduce the polarity of the compounds. The vial was then sealed using PTFE tape and held at 70°C for 1 h. Following this, the BSTFA + 1% TMCS was evaporated under a flow of nitrogen, and the entire sample was resuspended in 200 μl of ethyl acetate and sonicated for 10 min. Quantification was made using gas chromatography with flame ionization detection (Agilent Technologies, 6890) fitted with a low polarity column (Rxi-5HT, 15 m × 0.32 mm × 0.1 μm). The oven was held at 50°C for 2 min, before increasing by 10°C min^−1^ to 350°C. There, it was held isothermally for 10 min. The sample was then analysed using gas chromatography-mass spectrometry (GC-MS; Thermo Fisher Scientific, ISQ; Column - DB5-1HT, 15 m × 0.32 mm × 0.1 μm). The oven was held at 50°C for 2 min, before increasing by 10°C min^–1^ to 280°C. From there, the rate increased from 25°C min^–1^ to 380°C where it held isothermally for 5 min.

The data obtained were analysed using the software *Xcalibur* (Thermo Fisher Scientific), by comparison to spectra obtained from the NIST library. Apart from the triterpenoids, common compounds such as primary alcohols, fatty acids, alkanes and esters were accurately identified from their mass spectra. Owing to the large number of different plants in the study and the variety of possible triterpenoid structures, the triterpenoids were treated as a single group, and no effort was made to elucidate their exact structure. Compounds that could not be identified, based on GC-MS alone, were identified at the compound class level. If they were not identifiable at all, they were noted down as ‘unidentified’. For statistical analysis, we calculated the proportion of compound classes contributing to the total wax load of each species. We only considered compound classes separately if they were present in either the petals or leaves of at least seven species, and therefore calculated for each species the percentages of the following classes: aldehydes, acetate esters, alkanes, diketones, diols, esters, fatty acids, methyl alkanes, primary alcohols, secondary alcohols and triterpenoids, with a final class consisting of the unidentified compounds and the remaining identified compounds that were not from the classes already listed.

### 4.3. Scanning electron microscope

Plant tissue was collected in the wild and cut into suitable pieces, which were then stuck to a 12 mm SEM stub using conductive carbon discs. The stubs were left in a desiccation chamber until dry. Air drying is the method most frequently used for tissue preparation in the literature, and it is particularly useful for high-throughput work (e.g. [13]). While it introduces strong desiccation artefacts, this was not a problem for our study, owing to our focus on wax morphology alone. Following drying, the samples were sputter coated in 5 nm of gold prior to imaging in a Zeiss Evo 15 in vacuum mode. The stage fits eight SEM stubs at a time, which facilitates high-throughput work.

### 4.4. Statistical analysis

All statistical analysis was performed in R 4.3.0 [47]. The error is reported as standard deviation throughout the paper. All data and code, along with the electronic supplementary figures, are available on Figshare [48]. A core phylogeny for the 49 species used was constructed using *V.Phylomaker2* [49,50], using the GenBank Open Tree of Life from Smith and Brown [51].

The total wax coverage per cm^2^ was calculated using a known amount of tetracosane as internal standard. The differences in total wax coverage between the petals and leaves of species were compared using a phylogenetic paired *t*‐test [52] using *phytools* 1.5-1, after log transforming to meet assumptions of normality. The absence or presence of wax crystals was scored visually based on SEM images. Only structures clearly resembling those listed as crystals by Barthlott *et al*. [10] were considered crystals. We also considered the presence or absence of crystals on mean wax coverage, but because only three of the species had crystals on the petals, we separated this analysis from the phylogenetic paired *t*‐test to avoid introducing artefacts owing to the low sample size. Therefore, phylogenetic generalized least squares (GLS) for the crystal-based wax coverage (log transformed to meet normality assumptions) was conducted separately for leaves and petals using the core *gls* function, assuming a Brownian correlation matrix generated using *ape* 5.7.1 [53].

Analyses on the qualitative composition of the cuticular waxes were done on a compound class basis, considering the relative proportion of the compound class out of the total wax load. We explored whether the composition of the waxes was evolutionarily conserved by adapting the techniques developed by Perez-Lamarque *et al*. [54]. Although the package *ABDOMEN* was created for exploring phylosymbiosis in microbial gut communities, similar assumptions hold for how the chemical composition of wax may change over time, and if we assume that all the different compound classes could be present in vanishingly small quantities across the species, then the Brownian motion process considered by the authors is suitable for modelling the change in the chemical composition of the plant waxes. We used *ABDOMEN* to calculate Pagel’s λ, which indicates the influence of the evolutionary tree on the chemical composition of the species, where a value near zero indicates little or no effect of evolutionary history, and a value near 1 indicates that the evolutionary history of the species explains the change in composition well. Following the parameterization described by Perez-Lamarque *et al*. [54], model inferences were performed with four independent chains and 4000 iterations per chain that included a warm-up of 2000 iterations. Similarly, we followed Perez-Lamarque *et al*. [54] by calculating the significance of the λ by permutation analysis, where the calculated λ was compared to a distribution created from 100 sampled λ values taken from models where the compositional data were randomly shuffled across the plant species; the original λ was considered meaningful if it was greater than at least 95% of these randomly sampled values. We note that for the leaf data, 106 random samples were initiated (of which six failed to converge), while for the petal data, 103 random samples were initiated (of which three failed to converge).

We separately compared the percentage composition of the six main compound classes using phylogenetic paired *t-*tests, comparing the values for petals and leaves, as our earlier analysis demonstrated that the chemical composition of the petals was partially constrained by evolutionary history. Percentage compositions were log transformed to meet assumptions of normality, noting that the log-transformed data for esters still departed from normality owing to large outliers.

An ACL was calculated for the most common aliphatic compounds (present in at least half of the leaf samples: alkanes, aldehydes, esters, fatty acids and primary alcohols) using the formula for weighted arithmetic mean. The difference in non-transformed mean chain length between petals and leaves within species was compared using phylogenetic paired *t*-tests for the five compound classes separately, using only the species where the focal compound class was present in both the petal and leaf of the same plant to allow for direct comparisons.

## Data Availability

The supplementary data and R code is freely available at Figshare [48]. Electronic supplementary material is available online [55].
